# Characterization of TLR2, NOD2, and related cytokines in mammary glands infected by *Staphylococcus aureus* in a rat model

**DOI:** 10.1186/s13028-015-0116-0

**Published:** 2015-05-20

**Authors:** Heng Wang, Guangtao Yu, Hui Yu, Mingjie Gu, Jun Zhang, Xia Meng, Zongping Liu, Changwei Qiu, Jianji Li

**Affiliations:** College of Veterinary Medicine, Yangzhou University, Yangzhou, Jiangsu 225009 China; Jiangsu Co-innovation Center for Prevention and Control of Important Animal Infectious Diseases and Zoonoses, Yangzhou, Jiangsu 225009 China; College of Animal Science and Veterinary Medicine, Huazhong Agricultural University, Wuhan, Hubei 430070 China

**Keywords:** Cytokine, Innate immune reaction, NOD2, Rat mastitis, *S. aureus*, TLR2

## Abstract

**Background:**

*Staphylococcus aureus* causes subclinical mastitis as well as persistent and chronic infections in cattle. Bovine mastitis induced by *S. aureus* is often refractory to antibiotic treatment. Local innate immune defenses play an important role in eliminating the invading bacteria. TLR2 and NOD2 are important pathogen recognition receptors, but their functions have not been investigated in the context of early stages of mastitis. The present study examined TLR2, NOD2, and related cytokines in mammary glands infection induced by *S. aureus* at early stages in a rat mastitis model.

**Findings:**

All inoculated mammary glands developed mastitis. Acute changes were induced in mammary tissues infected with *S. aureus* at early stages and then chronic infections persisted until the end of the experiment. TLR2 and NOD2 mRNA expression increased significantly after inoculation with *S. aureus*. The expression levels of cytokine mRNAs, including TNF-α, IL-1β, IL-6, IL-10, and CXCL1, also increased. TGF-β1 expression was suppressed at early phase and IFN-γ mRNA expression increased significantly at a later stage.

**Conclusions:**

Mammary innate immune responses were activated after *S. aureus* inoculation. TLR2, NOD2, and inflammatory cytokines (TNF-α, IL-1β, IL-6, CXCL1, IL-10, TGF-β1, and IFN-γ) are involved in the response to mastitis induced by *S. aureus.*

**Electronic supplementary material:**

The online version of this article (doi:10.1186/s13028-015-0116-0) contains supplementary material, which is available to authorized users.

## Findings

*Staphylococcus aureus* causes infections in humans and animals [1, 2]. The pathogen often leads to subclinical bovine mastitis and tends to develop into persistent and chronic infections [3, 4]. Long-term infection causes reduced milk production, resulting in economic loss for dairy producers [5]. One possible mechanism of chronic infection is that the bacteria survive in the host phagocytes and some non-phagocytic cells, including mammary epithelial cells, where an effective concentration of antibiotics can not develop. Innate and acquired immune responses may also be not provoked effectively. *S. aureus* vaccines only have marginal benefit in alleviating the duration and severity of clinical symptoms [6]. Potential mechanisms of immune responses in mammary tissues are not well understood and immune suppression may exist.

Innate immunity is the first line of defense against pathogens and body injury, which is triggered by recognition of pathogen-associated molecular patterns (PAMPs) by pattern recognition receptors (PRRs) [7]. PRRs include Nod-like receptors (NLRs), which mediate cytosolic recognition of microbial molecules and promote their clearance [7–9], and Toll-like receptors (TLRs), which are located at the cell surface or within endosomal membranes and recognize a wide range of microbial molecules such as lipopolysaccharide (LPS), peptidoglycan, lipoteichoic acid, flagellin, and zymosan [8, 10, 11]. NOD2 senses muramyl dipeptide (MDP), which is a conserved structure in bacterial peptidoglycan (PGN) [12]. TLR2 recognizes lipoteichoic acid (LTA) and PGN from Gram-positive bacteria, and lipoproteins from Gram-negative bacteria [8, 13].

Experimental animal models are useful research tools to study mastitis [14]. Chandler [15] first reported experimental mastitis in a mouse model and mouse models have been employed to evaluate pathophysiology of mastitis [14, 16]. Rat models were introduced to study mastitis because larger teat channels facilitate bacterial inoculation [17]. The present study aims to reveal the characteristics of TLR2, NOD2, and related cytokines in mammary glands against mastitis induced by *S. aureus* at an early stage in a rat mastitis model.

*S. aureus* (YZ20108) was previously isolated and identified from a dairy cow with persistent and recurrent mastitis. The bacteria were cultured in Luria-Bertani broth (LB) at 37 °C and harvested at log phase. The number of colony-forming units (CFU) was determined by serial dilution and plate count method.

Pregnant Wistar rats, n = 84, weighing 275 ± 25 g, were purchased from the Laboratory Animal Center of Yangzhou University, China. All the rats were raised in plastic cages with sterilized saw dust, temperature of 23 ± 2 °C and relative humidity of 50 ± 5 %. They were fed with commercial diet and had free access to water *ad libitum*. Rats were randomly divided into two groups: experimental group (n = 42) and control group (n = 42). L4 and R4 abdominal mammary glands of rats in experimental group were inoculated with 0.1 ml *S. aureus*, containing 2 × 10^7^ CFU/•ml on the 4th day after parturition while the control group with the same volume of physiological saline. The outline of inoculation is as follows: rats were anesthetized with 2 % pentobarbital sodium solution by intraperitoneal injection (0.2 ml/100 g body weight). After anesthesia, the body surface was cleaned and disinfected. Then a 33-gauge needle equipped with 1 ml syringe was inserted into the mammary duct of L4 or R4 and 0.1 ml *S. aureus* or sterile physiological saline was administrated to experimental or control rats, respectively. six rats from each group were euthanized with by cervical dislocation after anesthesia by 2 % pentobarbital sodium injection prior to inoculation (0 h) and then at 6, 12, 24, 48, 72, and 96 h post inoculation (pi). Mammary tissue samples were aseptically collected and prepared for bacterial counts, histopathological examination, and molecular analyses. All the experiments were conducted in accordance with the Guide for the Care and Use of Laboratory Animals of the National Research Council. The animal care and use committee of Yangzhou University approved all experiments and procedures.

To quantify the level of infection in mammary glands, mammary tissues were aseptically collected, weighed, and homogenized with sterile physiological saline (1:10, W:V). Suspensions were centrifuged at 10 000 rpm for 5 min at 4 °C to discard fat and supernatant. Sediments were suspended and shaken thoroughly. Homogenates were serially diluted in physiological saline, plated on nutrient agar containing 5 % sheep blood and cultured at 37 °C. Bacterial CFU was counted and the level of infection was estimated by CFU per 100 mg of mammary tissue.

Mammary tissues were collected at 0 (prior to inoculation), 6, 12, 24, 48, 72, and 96 h pi and fixed in 10 % neutral formalin for histopathological examination. Fixed tissues were embedded in paraffin and cut into 5 μm continuous sections and stained by hematoxylin-eosin (H&E).

The extraction of total RNA and real-time fluorescence quantitative polymerase chain reaction (PCR) reaction were performed as previously described [11]. The sequences of primers are listed in Table 1. The reaction was conducted in triplicate for each sample and the mean value was used to calculate mRNA expression levels. Six samples of each group were measured at each time point. The fold changes for gene expression were calculated with the relative quantification method and *Gapdh* was applied as a housekeeping gene. The average dCt of samples collected at 0 h was used as the calibrator for each sample.

**Table 1:** Sequences, amplification product size, and GenBank accession number of amplification genes of rats

Gene	Primer sequence (5ʹ to 3ʹ)	Product (bp)	Accession number
Gapdh	F:CCAGCAAGGATACTGAGAGCAA	101	NM_017008.4
	R:GGATGGAATTGTGAGGGAGATG		
TLR2	F:CAAACTGGAGACTCTGGAAGCA	120	NM_198769.2
	R:AGGTAGCTGTCTGGCCAGTCA		
NOD2	F: ACAAAGACGCCGACACTATACTG	241	NM_001106172.1
	R: TCAAGGAGGAACTGGAAGACG		
TNF-α	F: GTAGCCCACGTCGTAGCAA	217	NM_012675.3
	R: AAGTGGCAAATCGGCTGAC		
IL-1β	F:GCAATGGTCGGGACATAGTT	152	NM_031512.2
	R:GACTTGGCAGAGGACAAAGG		
IL-6	F: CACAAGTCCGGAGAGGAGAC	168	NM_012589.2
	R: ACAGTGCATCATCGCTGTTC		
CXCL1	F:GGCGGAGAGATGAGAGTCTG	182	NM_030845.1
	R:AGGCATTGTGCCCTACAAAC		
IL-10	F:CACTGCTATGTTGCCTGCTCTTACT	73	NM: _012854.2
	R: TTATTGTCACCCCGGATGGA		
TGF-β	F:CAACAATTCCTGGCGTTACCTT	121	NM_021578.2
	R:CTGTATTCCGTCTCCTTGGTTCA		
IFN-γ	F: AGGAACTGGCAAAAGGACG	196	NM_138880.2
	R: CGAACTTGGCGATGCTCAT		

All statistical analyses were performed using IBM SPSS Statistics 20 (Japan). Data was expressed as mean ± SE except the bacterial count in mammary tissue, which was converted to log_10_ to keep a normal distribution for statistical analyses. Differences were considered significant at *P* < 0.05 analyzed by ANOVA and followed by Bonferroni *post hoc* test.

**Fig. 1:**
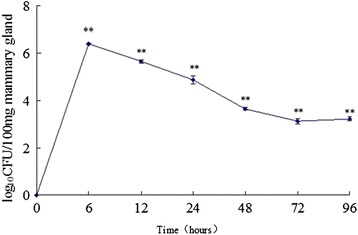
Bacterial counts change in mammary tissues from experimental group inoculated with *S. aureus* by 2 × 10^6^ CFU at different time points. Data were shown by log_10_ colony-forming units per 100 mg ± (SE) of mammary tissue. **P < 0.01

The results of bacterial counts showed that *S. aureus* were present in all inoculated mammary glands throughout the study. The quantity of *S. aureus* in the mammary glands peaked at 6 h pi and then decreased gradually until 96 h pi (Fig. 1). Bacterial colonization in mammary tissues was not found in control group.

**Fig. 2:**
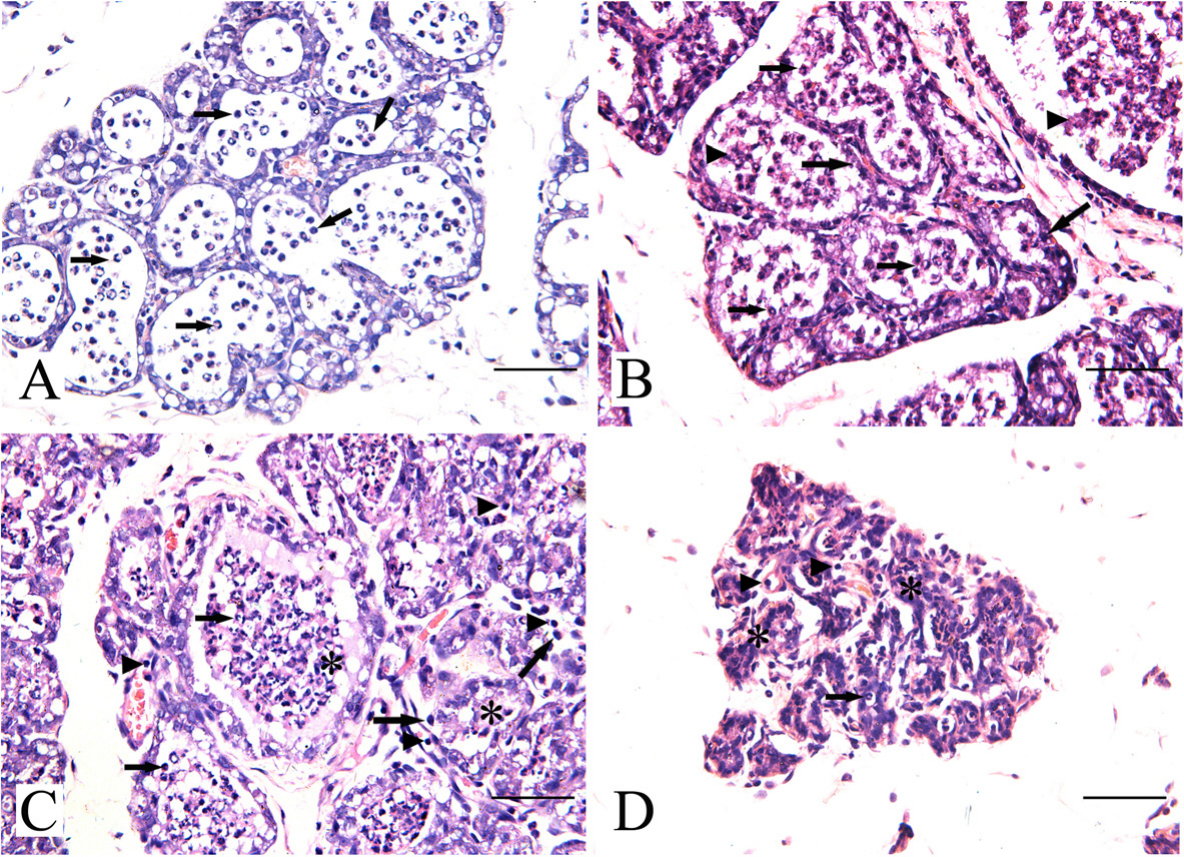
Histopathological findings of mammary glands inoculated with *S. aureus*. H&E stain. Bar = 50 μm. **a** PMNs predominately neutrophils appeared in the intralobular ducts, alveoli, and interlobular connective tissues at 6 h pi (arrow). **b** Secretory units (arrowhead), epithelial cells debris (large arrow) mixed with neutrophils (small arrow) in acini at 12 h pi. **c** Increased number of neutrophils (small arrow), lymphocytes (arrowhead), and plasma cells (large arrow) appeared in the mammary alveoli, accompanied with epithelial cells, and casein (asterisk) at 24 h pi. **d** Atrophy of acini (asterisk) with netrophils (arrow) and lymphocytes (arrowhead) in the mammary alveoli and intralobular connective tissues at 48 h pi

In experiment group, histopathological examination revealed enlarged mammary alveoli and appearence of polymorphonuclear cells (PMNs) in the intralobular ducts, alveoli, and interlobular connective tissues at 6 h pi (Fig. 2a). PMNs infiltrated into the mammary alveoli at 12 h pi, along with secretory units, round concretions of casein, and cellular debris. Destruction of epithelial cells was seen in some acini (Fig. 2b). At 24 h pi, inflammation had further developed, and more neutrophils, lymphocytes, and plasma cells appeared in the mammary alveoli and intralobular connective tissues, accompanied with epithelial cells damage and round concretions of casein in alveoli (Fig. 2c). Mammary tissue structure was damaged and alveoli developed atrophy, with inflammatory cells distributed in the alveoli at 48 h pi (Fig. 2d). At 72 h and 96 h, inflammatory cells decreased gradually, mammary alveoli atrophied. No pathological changes were observed in the control group (Additional file 1).

**Fig. 3:**
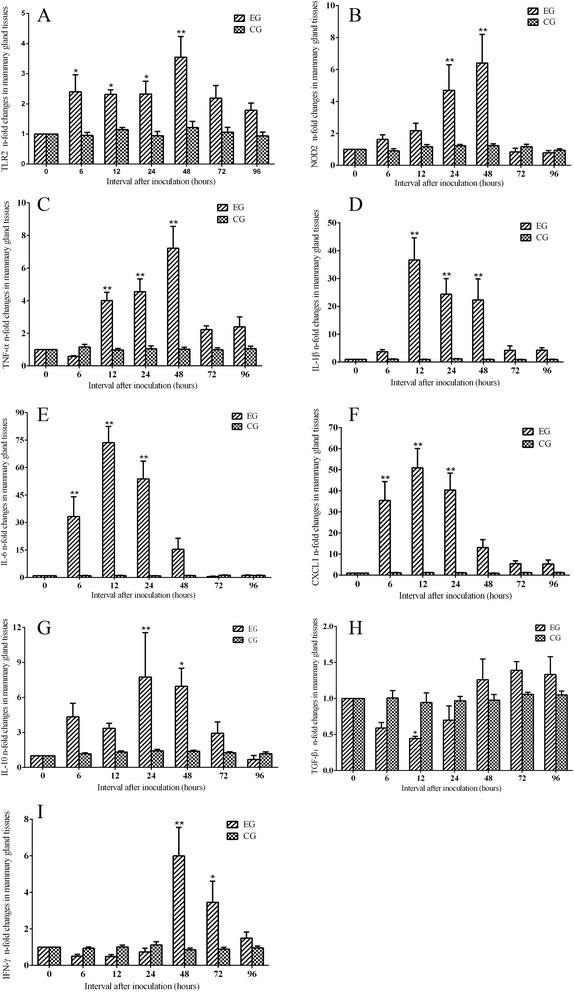
Fold changes (n-fold) of (**a**) TLR2, (**b**) NOD2, (**c**) TNF-α, (**d**) IL-1β, (**e**) IL-6, (**f**) CXCL1, (**g**) IL-10, (**h**) TGF-β1, and (**i**) IFN-γ mRNA expression in the mammary tissue of rats after intramammary inoculation with *S. aureus* (experimental group, EG), or PBS (control group, CG). Statistically significant differences between the experimental group and control group are indicated (*P < 0.05, **P < 0.01)

Intramammary inoculation with *S. aureus* elicited significant changes in the mRNA levels of TLR2, NOD2, and some cytokine genes in mammary glands of experimental group. Compared to pre-inoculation levels of mammary samples at 0 h, the TLR2, NOD2, and TNF-α mRNA levels of mammary samples in experimental group increased gradually at 12 and 24 h pi, peaked at 48 h pi and declined at 72 and 96 h pi (Fig.3a- c). TLR2 is a crucial immune recognizable receptor activated by *S. aureus* infection in mammary tissue [18, 19]. Our study supports the hypothesis that TLR2 plays an important role at the early inflammation induced by *S. aureus* in mammary glands. Meanwhile, PMNs migrated into the mammary tissues, which may enhance the ability of clearing the bacteria. We also find an association of PMNs migration into the mammary tissues with the higher expression of NOD2, which may contribute to enhancing innate immune response and accelerate elimination of bacteria. The higher NOD2 expression in the mammary tissues is an indication of the *S. aureus* recognition and immune response against the pathogen*.* TNF-α early up-regulation is crucial for the prompt defense against invading pathogens. IL-1β plays an important role in host immune reaction by taking part in inducing neutrophil recruitment to control the *S. aureus* infection [20]. In our study, mRNA level of IL-1β also increased sharply after inoculation 6 h pi and peaked at 12 h pi (Fig. 3d), which was in accord with histological examination characterized by plenty of PMNs infiltrating the mammary glands. The mRNA levels of IL-6 and CXCL1 were up-regulated significantly and peaked at 12 h pi and then decreased gradually at 24 and 48 h pi (Fig. 3e and f). IL-6 is a pleiotropic cytokine, which involved in inflammatory responses, differentiation, activation of lymphocytes, and production of immunoglobulins [21]. It is supposed that a swift and strong expression level of IL-6 mRNA induces migration of PMNs and triggers the activation of transcription including TNF-α and IL-1β. The mRNA expression of CXCL1, analog of IL-8 [22], increased dramatically after inoculation. It suggested that CXCL1 was activated and could attract PMNs migration into the inflammatory location and eliminate the invading pathogens. In addition, gene expression of TNF-α, and IL-1β increased more slowly than CXCL1 in mammary tissues, which suggested that infiltrated PMNs contributed to the production of TNF-α and IL-1β after CXCL1 activation. The expression of IL-10 mRNA in mammary samples increased significantly at 24 and 48 h pi (Fig. 3 g). However, the transcriptional level of TGF-β1 dropped sharply at 12 h pi in experimental group and then increased slowly (Fig. 3 h). It suggested that TGF-β1 was suppressed at the initial phase of mammary infection*.* IFN-γ is a cytokine that is very important for innate and adaptive immunity against infection of virus and intracellular bacteria [23]. The mRNA expression of IFN-γ decreased slightly at 6 and 12 h pi, but increased dramatically at 48 h pi and then declined gradually at 72 and 96 h pi (Fig. 3i). It revealed that elevate expression of IFN-γ in later stage was probably related with *S. aureus* invasion and survival in the tissue cells as time went on, and had immunostimulatory, and immunomodulatory effects.

## Conclusion

The local innate immune response of mammary glands was swiftly activated after *S. aureus* inoculation during the initial stage of infection, characterized by up-regulation of gene expression of TLR2, NOD2, TNF-α, IL-1β, IL-6, and CXCL1. Additionally, anti-inflammatory cytokine IL-10 took part in the inflammation modulation and TGF-β1 was suppressed during the *S. aureus* infection. Considerable quantity of *S. aureus* that survived in the mammary tissues led to persistent inflammation, and IFN-γ played a role in the later inflammation.

## Additional file

Additional file 1:
**Microphotographs of mammary glands inoculated with physiological saline (A, B).** H&E stain. Bar = 50 μm.

## Abbreviations

CFU: Colony-forming units
IFN: Interferon
IL: Interleukin
LPS: Lipopolysaccharide
LTA: Lipoteichoic acid
MDP: Muramyl dipeptide
NLRs: Nod-like receptors
PAMPs: Pathogen-associated molecular patterns
PCR: Polymerase chain reaction
PGN: Peptidoglycan
PMNs: Polymorphonuclear cells
PRRs: Pattern recognition receptors
TGF-β: Transforming growth factor-β
TLRs: Toll-like receptors
TNF-α: Tumor necrosis factor-α
