# Supplementary material for: Characterization of TLR2, NOD2, and related cytokines in mammary glands infected by Staphylococcus aureus in a rat model
Source: Acta Vet Scand. 2015 May 20;57:25. doi: 10.1186/s13028-015-0116-0 (PMC4672474; doi:10.1186/s13028-015-0116-0)

Additional file 1. Microphotographs of mammary glands inoculated with physiological saline (A, B). H&E stain. Bar=50μm.


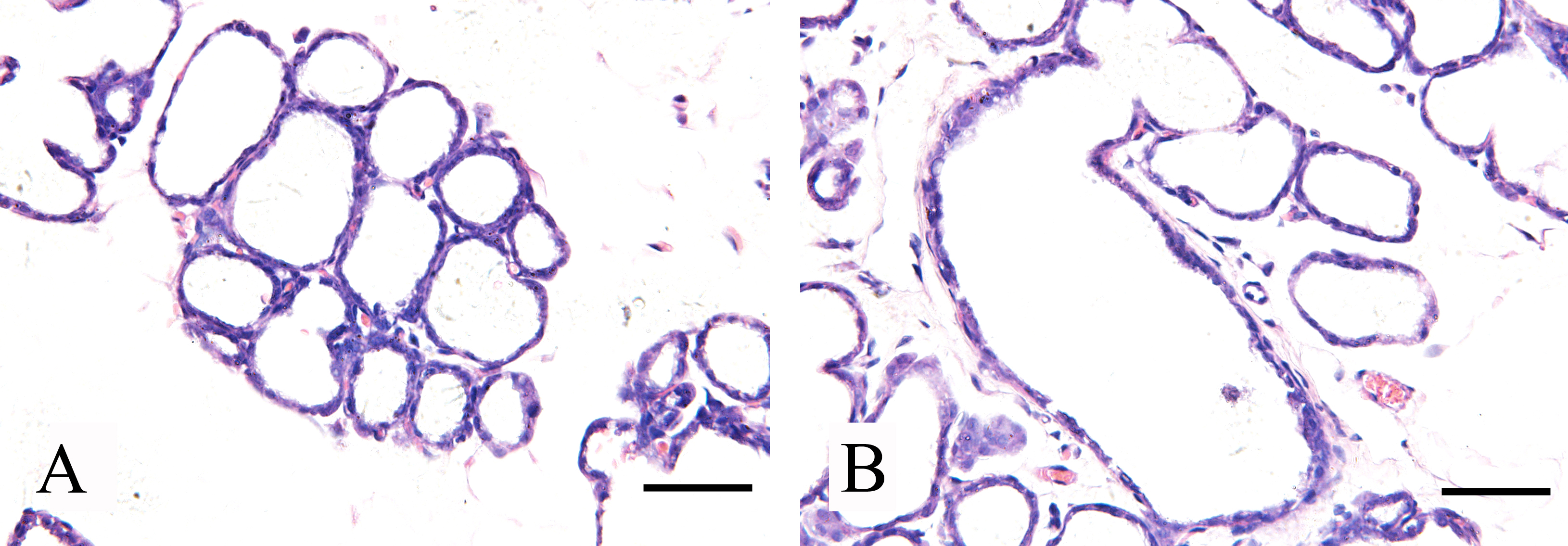

Supplement: Additional file 1: — Microphotographs of mammary glands inoculated with physiological saline (A, B). H&E stain. Bar = 50 μm. [file 13028_2015_116_MOESM1_ESM.doc]
